# Development of a Transparent Interactive Decision Interrogator to Facilitate the Decision-Making Process in Health Care

**DOI:** 10.1016/j.jval.2010.12.002

**Published:** 2011-07

**Authors:** Sylwia Bujkiewicz, Hayley E. Jones, Monica C.W. Lai, Nicola J. Cooper, Neil Hawkins, Hazel Squires, Keith R. Abrams, David J. Spiegelhalter, Alex J. Sutton

**Affiliations:** 1 Department of Health Sciences, University of Leicester, Leicester, UK; 2 Department of Social Medicine, University of Bristol, Bristol, UK; 3 Oxford Outcomes, Oxford, UK; 4 Centre for Health Economics, University of York, York, UK; 5 School of Health and Related Research, University of Sheffield, Sheffield, UK; 6 Centre for Mathematical Sciences, University of Cambridge, Cambridge, UK

**Keywords:** bias adjustment, decision model, interactive, meta-analysis, RExcel, software, TIDI

## Abstract

**Background:**

Decisions about the use of new technologies in health care are often based on complex economic models. Decision makers frequently make informal judgments about evidence, uncertainty, and the assumptions that underpin these models.

**Objectives:**

Transparent interactive decision interrogator (TIDI) facilitates more formal critique of decision models by decision makers such as members of appraisal committees of the National Institute for Health and Clinical Excellence in the UK. By allowing them to run advanced statistical models under different scenarios in real time, TIDI can make the decision process more efficient and transparent, while avoiding limitations on pre-prepared analysis.

**Methods:**

TIDI, programmed in Visual Basic for applications within Excel, provides an interface for controlling all components of a decision model developed in the appropriate software (e.g., meta-analysis in WinBUGS and the decision model in R) by linking software packages using RExcel and R2WinBUGS. TIDI's graphical controls allow the user to modify assumptions and to run the decision model, and results are returned to an Excel spreadsheet. A tool displaying tornado plots helps to evaluate the influence of individual parameters on the model outcomes, and an interactive meta-analysis module allows the user to select any combination of available studies, explore the impact of bias adjustment, and view results using forest plots. We demonstrate TIDI using an example of a decision model in antenatal care.

**Conclusion:**

Use of TIDI during the NICE appraisal of tumor necrosis factor-alpha inhibitors (in psoriatic arthritis) successfully demonstrated its ability to facilitate critiques of the decision models by decision makers.

## Introduction

Decision-making systems in health care are increasingly designed in such a way to ensure equity of access and to optimize the use of limited health care resources, and this approach has been adopted now in many countries. To aid the decision-making process, health technology assessments (HTAs) are performed that evaluate both the effectiveness and cost-effectiveness of new technologies compared to existing technologies, resulting in guidance to national health care services. A significant component of HTAs is the economic evaluation that often relies on the development of elaborate decision analytic models [1,2]. Such models require a large number of inputs (related to cost, clinical effectiveness, natural disease history, and/or quality of life), some of which may be obtained from primary data collection, but more often rely on the re-analysis of published or other secondary data [3].

Historically, a two-part approach to HTA has been adopted, where individual parameter estimates are first obtained either directly or by conducting preliminary analyses (e.g., a meta-analysis where multiple sources of evidence exist) and then extracted and input into a decision model, often assuming independence and parametric distributions [4]. More recently an integrated one-step approach has been advocated [4,5] that unifies the two stages described above where all preliminary analyses and the decision model are conducted within a single analytical framework. The main advantages of this integrated approach over a two-stage approach include relaxing the assumptions of independence and parametric distributions, and the facilitation of transparency, sensitivity analysis, and updating. To date this has been achieved by programming all analysis components and evaluating them within a single statistical program. Markov Chain Monte Carlo simulation, as implemented in the WinBUGS package [6], provides an ideal environment for this. However, despite the advantages of such an approach, limitations include 1) lack of a user-friendly interface, and 2) very limited numerical and graphical output facilities. This makes it very difficult for models constructed in this way to be interrogated and fully appraised by non-technical experts including decision makers.

Decision making on new health care technologies in England and Wales is conducted by the National Institute for Health and Clinical Excellence (NICE). Health technology appraisal documents are produced both by independent academic teams and manufacturers, for consideration by NICE appraisal committees. Having carefully appraised these documents, the committees have to make decisions based on their own informal judgments about the evidence, uncertainty, and assumptions made [7,8]. Often the appraisal documents contain decision analytic models that are probabilistic in nature and thus reflect parameter uncertainty to some degree. However, usually uncertainties exist beyond those quantified in this way (e.g., parameters for which no data exist, structural uncertainties in the decision models). Hence, sensitivity analysis is an important part of the decision-making process and is used to investigate the robustness of the model results across different scenarios. This usually entails analysts anticipating, running, and reporting all possible scenarios that might be of interest to decision makers prior to their discussions. However, where all scenarios of interest have not been anticipated, this can lead to an inefficient process of repeated evaluations as analysts respond to sequential requests from decision makers (or decisions are made without the opportunity to formally conduct the relevant analyses). Therefore, it would be advantageous to allow decision makers, such as the appraisal committees of NICE, to run analyses under different scenarios, ideally in real-time during actual committee discussions.

In this report we introduce the framework concept of a transparent interactive decision interrogator (TIDI) together with an illustrative example implementation. This enhances the integrated one-stage approach discussed above, by incorporating a user interface to control many aspects of the modeling (i.e., preliminary analyses and evaluation of the decision model). It has been designed to help to overcome problems of interpretation, clarity and transparency in the decision process by facilitating critiques of the model structure, assumptions and uncertainty by a broad spectrum of people ranging from the analysts themselves when developing the model through to non-technical decision makers and other stakeholders. TIDI has been developed to be used in real-time by committees such as those at NICE during their deliberations. Hence, TIDI can make the process of evaluating uncertainty more transparent, faster, and more efficient; while at the same time avoiding arbitrary limitations of pre-prepared analyses of a restricted number of scenarios that might compromise its validity. A further technical advancement made possible by this approach is a relaxation of the need for all components of the modeling to be conducted in a single piece of code using MCMC methods. As we describe in more detail below, the TIDI interface can control multiple separate modeling components conducted in packages such as (but not restricted to) R and WinBUGS and coordinate them in such a way to maintain a one-step approach to analysis. This allows the utilization of the strengths of each package while overcoming their individual limitations.

The remainder of this article will 1) describe how TIDI works at a conceptual level; 2) introduce a range of features that can be implemented through an illustrative example decision model; and 3) provide a brief account of our experience piloting the interface at a real NICE technology appraisal committee meeting. The discussion will provide some closing remarks.

While we don't intend this article to present a software tutorial on “how to program a TIDI interface,” we do provide all code developed for the illustrative example. The instructions on how to use the interface and how to install necessary software are included in Appendix A, which can be found at doi:10.1016/j.jval.2010.12.002.

## Methods

### Developing TIDI

There are a number of software packages available to implement health economic models. Although Excel (Microsoft Corporation, Redmond, WA) is mostly accessible and known to a wider community of nonexperts, decision models designed using Excel spreadsheets tend to be incomprehensible by the nature of the way they are constructed on an underlying spreadsheet. There have also been reports of Excel built-in statistical functions and procedures being faulty [9] or based on nonstandard rules of operator precedence [10]. Hence, we wished to keep the familiarity of Excel and the flexibility it allows for developing graphically appealing and intuitive “point and click” front-end control panels, while implementing the decision model and any subsidiary analyses (such as meta-analyses, modeling of individual level data sets, etc) in packages more suited for these tasks. Thus, TIDI's decision models have been developed in the specialized statistical packages R [11] and/or WinBUGS [5] as we believe these are clearer, more flexible, transparent, and computationally efficient, as well as allowing analysis methods not possible in Excel such as network meta-analysis [12]. However, the use of these specialized packages is entirely “behind the scenes”: the Excel-based user interface allows decision makers to have access to features of all components of a decision model developed in R and WinBUGS without a need of knowledge of these software packages. This Excel front-end not only makes it possible to change the assumed values or distributions of the model parameters and re-run models under different scenarios in real time, but can also provide a control over the model assumptions. TIDI can also provide interactive access to supplementary analyses, for example influence analysis that can help to establish which of the parameters have the most impact on the cost-effectiveness estimates, and the meta-analyses carried out to estimate the efficacy parameters. A specific meta-analysis module of TIDI can enable interactive inclusion/exclusion of studies as well as bias adjustment [13] in real time for any such analyses contributing to decision model parameter estimation.

An Excel-based interface, programmed in Visual Basic for applications (VBA; Microsoft Corporation) [14,15], is at the center of TIDI. This allows a range of assumptions to be changed by using graphical controls set out on the Excel spreadsheet. RExcel [16,17], which is an add-in to Excel, provides communication between Excel and R. All data used by the model components can be stored in the Excel spreadsheets and transferred to the R workspace and various actions, for example execution of the decision model or its components, can also be activated using controls located on the Excel spreadsheet. Results of the decision model are then transferred from R back to Excel via RExcel. R also has powerful and flexible graphical capabilities, and output can be represented graphically in Excel using custom plots created “on the fly” in R (a tutorial on how to construct a simple Excel interface linked to R code can be found in the RExcel manExcel interface linked to R codeual with “demo” examples provided by the authors of RExcel with the download of the software).

**Fig. 1:**
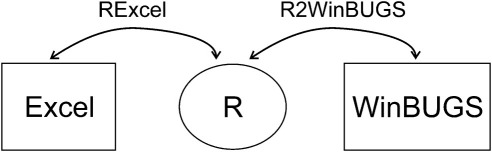
Transfer of data and commands between the software packages.

Having this possibility of running programs developed in R from Excel allows the user to take this further and to execute (from Excel) additional model components, including complex evidence syntheses developed in WinBUGS [18]. This is achievable by using the R2Winbugs package available in R [19]. R2WinBUGS provides additional R commands that make it possible to specify (in the R code) all data and parameters necessary for the WinBUGS code to run. In this way, using RExcel, data required to run WinBUGS code can be passed from the Excel spreadsheet to the R workspace from which R code (run from Excel via RExcel) sends it to WinBUGS together with the instructions on how to process these data. This framework for the transfer of data and commands between different pieces of software is shown schematically in Figure 1. An important principle in this approach is that it is necessary to keep estimation of correlated parameters within the same analysis component. If this principle is not adhered to then the correlations between parameters will be lost within the evaluation of the decision model.

It is important to note that it would be possible to connect further packages into a TIDI interface. For example, routines are available that allow SAS (SAS Institute, Cary, NC) and Excel to communicate [20], and Stata 10 (StataCorp LP, College Station, TX) allows direct exportation to EXCEL format (and communication between STATA and WinBUGS has been developed [21]). Generally, any package that can be controlled using a scripting language (or already has an Excel interface, such as TreeAGE [TreeAge Software, Inc., Williamstown, MA]) can be used. Since HTAs are often conducted by multidisciplinary teams, and specialists may have different software preferences and knowledge, we believe the creation of a TIDI type control panel in Excel may have the added benefit of “harmonizing” analysis components.

### Illustrative example: Routine antenatal anti-D prophylaxis for Rhesus D-negative pregnant women

We present an illustrative TIDI application that was implemented for an economic decision model developed for the NICE appraisal of routine antenatal anti-D prophylaxis (AADP) for Rhesus D (RhD)-negative pregnant women [22]. RhD-negative women carrying an RhD-positive fetus can develop anti-D antibodies (RhD sensitization process) that can affect subsequent pregnancies, potentially leading to hemolytic disease of the newborn. Anti-D prophylaxis is used to reduce the incidence of sensitization. The decision model evaluated whether four anti-D treatments are cost-effective when administered to RhD-negative pregnant women who are in their first pregnancy or to all RhD-negative pregnant women.

This example has also been used by Turner et al. [13] to develop methods for modeling bias in evidence synthesis, where authors used the effectiveness data from the first NICE appraisal of anti-D prophylaxis [23]. These methods have been implemented in TIDI as part of its interactive meta-analysis module to illustrate how TIDI brings such cutting-edge approaches to analysis within the grasp of non-technical experts.

## Interface capabilities

### Constructing a model scenario

**Fig. 2:**
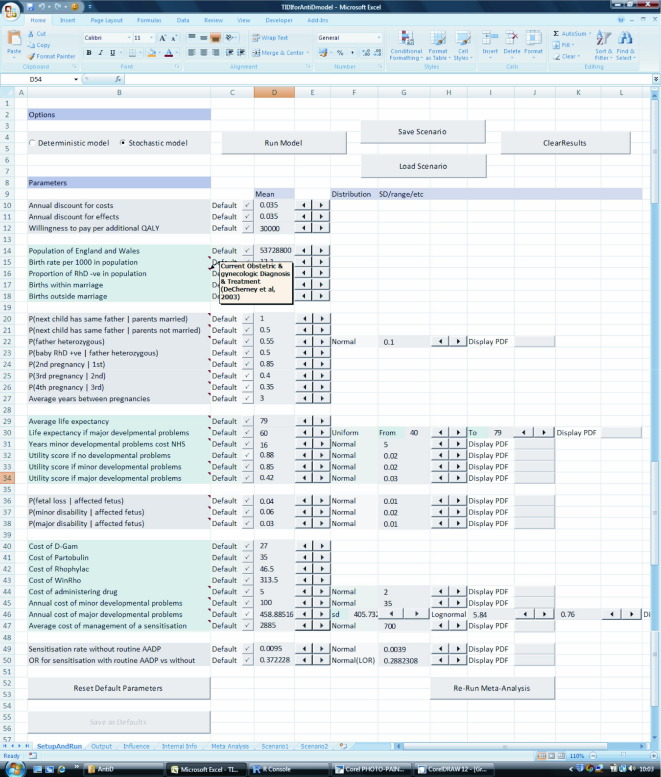
The front page of TIDI with setup of the model parameters.

The controls for the model parameters and actions are set out on the front Excel worksheet of the TIDI control interface, called “SetupAndRun.” These controls allow assumptions about the values and associated uncertainty of the parameters informing the decision model to be changed (see elsewhere for a full description of the model and its assumptions [22]). Figure 2 shows the layout of these controls. The controls can be designed to allow the user to switch between alternative options for modeling the cost-effectiveness. For example, in this interface the “radio” buttons enable switching between a deterministic or stochastic (i.e., incorporating uncertainty) model. This approach potentially allows the user to have access to the model parameter values and model structure, assumptions, and leads to building new model scenarios.

### Decision model execution and results

**Fig. 3:**
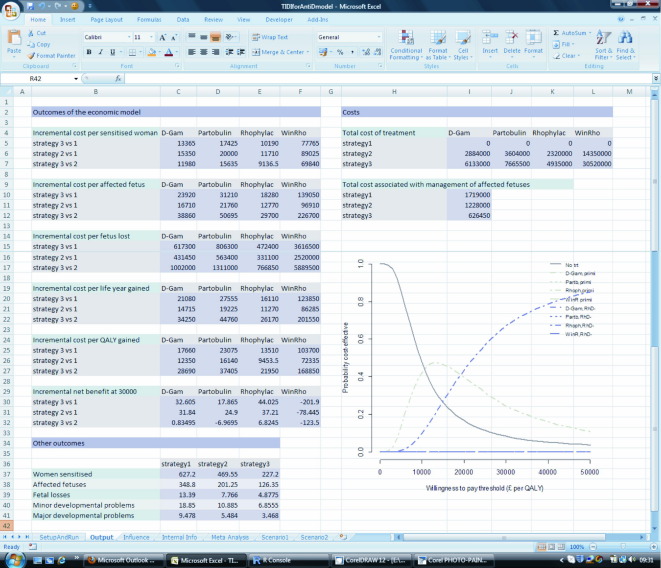
The output page of TIDI displaying all results of the decision model and acceptability curves plot.

Once a new model scenario has been built, the model can be re-run by pressing the “Run Model” button. All model parameter values are then loaded into an R workspace where the R2WinBUGS commands are used to send data to and execute code in WinBUGS (in this example the decision model is programmed in WinBUGS); then the outcome information is sent back to the R workspace. The range of outcomes for this model consists of costs, incremental costs (per sensitized woman, affected fetus, fetus lost, life year gained, and quality-adjusted life year gained), incremental net monetary benefit (INMB) and other clinical outcomes. The results of the model are automatically transferred into the “output” worksheet via RExcel. As shown in the example in Figure 3, the “output” spreadsheet lists costs, outcomes of the economic model, and other outcomes of clinical interest for strategies 1–3 (strategy 1: control group, conventional management; strategy 2: AADP to primigravida only; strategy3: AADP to all RhD-negative pregnant women). For the stochastic model, a graph showing the cost-effective acceptability curves [24] is constructed in R but displayed in Excel. This shows the probabilities of each treatment and strategy combination being cost-effective compared to the alternatives, under a range of willingness to pay thresholds.

Additionally, the interface provides various ways for storing information, for example, on origin of the parameters' estimates used in the model. Also, full sets of data corresponding to different scenarios can be stored and accessible in an interactive manner as described in Appendix B, which can be found at doi:10.1016/j.jval.2010.12.002.

### Meta-analysis module

In this model, effectiveness estimates (odds ratio [OR] here are all interventions assumed to have the same effectiveness [22]) that inform the decision model are obtained from a meta-analysis. The developed meta-analysis module of TIDI allows the user to have access to many details of the analysis and to make some adjustments to the evidence synthesis. The meta-analysis interface is designed in such a way to enable selection of any subset of available studies for which pooled outcomes can be recalculated. For example, if a committee member raises doubts about the quality or relevance of a particular study, it is easy to exclude this study and re-run the meta-analysis from the Excel front end. The resulting estimates are then displayed in tabular form as well as using interactive forest plots. The outcome of the new meta-analysis (OR) can then be uploaded into the front worksheet containing the model parameters and the decision model can be re-run with the amended effectiveness estimates.

This sensitivity analysis can be vital in situations when some of the available studies are, for example, of questionable quality or do not represent the considered population of patients. However, we go beyond this simple sensitivity analysis and allow for adjustment of results from individual studies in the meta-analysis for the various potential internal and external biases as those considered by Turner et al. [13], based on information from expert elicitations and using a Bayesian method. The interface enables the user to choose which types of bias to adjust for. The user can decide whether or not to adjust for internal biases reflecting the lack of rigor of studies included in the meta-analysis, or for external biases that relate to the relevance of those studies included, such as their short comings in answering the target question. The decision maker can also decide whether (within those internal and external biases) to adjust only for biases that act additively or for those believed to be proportional to the effect of the intervention. In addition TIDI enables selection of the specific bias assessors to be included in the bias-adjusted meta-analysis, allowing sensitivity to which expert opinions are incorporated to be assessed.

**Fig. 4:**
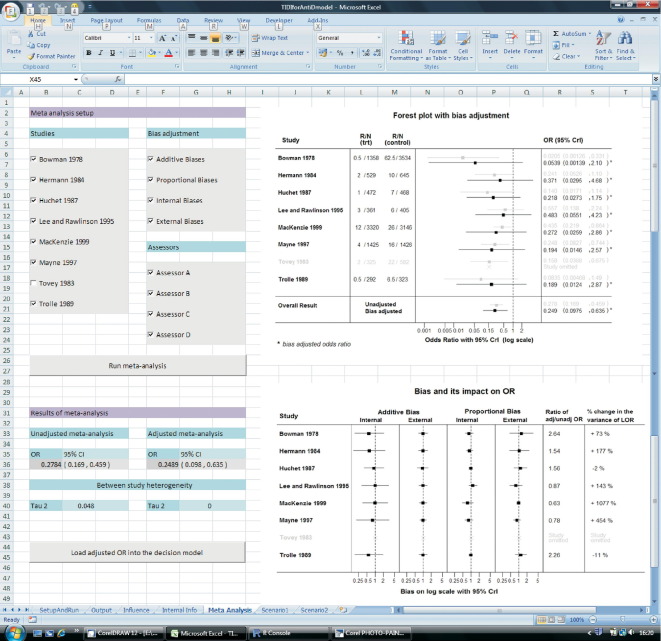
Meta-analysis module of TIDI with bias adjustment options.

Figure 4 displays the front end of the meta-analysis module and the tick boxes for study, bias type, and assessor selection. The results are provided in tabular form and also with interactive forest plots. After selecting the studies, types of biases and the bias assessors to be included, the meta-analysis can be re-run (by pressing the button “Run meta-analysis”) and updated estimates are then displayed in the updated forest plots in which the results of the unadjusted meta-analysis are also displayed (grayed-out) for comparison. In the lower “forest plot” the impact of the included bias on the meta-analysis estimates is displayed in more detail for information. Pressing the button “Load adjusted OR into the decision model” sends the new estimate of the OR to the front spreadsheet of TIDI (“SetupAndRun”) and replaces the original value with the new estimate, which can then be used in a new scenario of the decision model.

### Influence analysis module

**Fig. 5:**
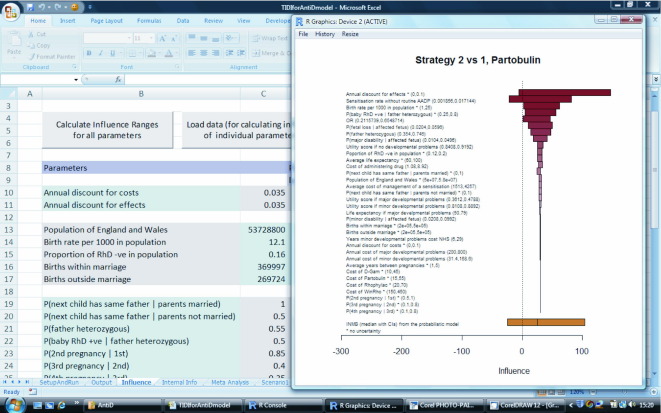
Example of tornado plot for Partobulin and comparison of strategy 2 versus 1.

Health economic decision models can be very complex, being informed by a large number of inputs related to the cost and effectiveness of treatments. It is infeasible to carry out a sensitivity analysis that explores all possible scenarios considering different values of all parameters. Influence analysis can help to identify those parameters that have the highest impact on the cost-effectiveness estimates such as INMB or incremental cost-effectiveness ratio (ICER), and can be represented by means of tornado plots [25]. Figure 5 shows an example of the (deterministic) tornado plot for INMB calculated for Partobulin, comparing strategies 2 and 1. The parameters most influential for this INMB (indicated by the widest bars at the top of the plot) are the annual discount for effectiveness and the sensitization rate without routine AADP (i.e., control group event rate). The bottom bar shows the INMB with 95% CI obtained from the stochastic model (for the base case set of parameters) for comparison. Further details on tornado plots and their stochastic counterparts can be found in Appendix B at doi:10.1016/j.jval.2010.12.002.

## Use of TIDI in practice

TIDI has also been developed for an economic model (and associated evidence syntheses) commissioned by NICE assessing the cost-effectiveness of tumor necrosis factor alpha (TNF-alpha) inhibitors (etanercept, infliximab, and adalimumab) when used in treatment of psoriatic arthritis [26]. This interface was used by the NICE technology appraisal committee during their meetings to support their decision-making process. We have not been able to present this TIDI application in this report for reasons of confidentiality. It was created for a more complex model (compared to the decision model considered here) and it gave opportunity to explore, for example, alternative ways of modeling utility, the impact of alternative stopping rules in the Markov model, or subgroup analyses. The meta-analysis module of this interface contained multiple evidence syntheses including network meta-analyses. Also, in addition to output of the decision model included in the Anti-D example above, a plot for marginal cost effectiveness of the various alternative interventions was constructed. During the committee meetings, the interface was used—in real time—to answer some queries from the committee members about the assumptions regarding clinical effectiveness of the interventions and their impact on the cost-effectiveness estimates.

For this technology appraisal, the assessment group carried out cost-effectiveness analysis across 31 sensitivity analysis scenarios [27]. It covered many aspects of the modeling in terms of the parameter values and structure of the model. Despite this very comprehensive analysis, the committee members still had questions about some of the model assumptions and their effect on the estimates from the economic decision model. To answer these questions, additional scenarios beyond those presented in the pre-prepared analysis were required. The scenarios, considered during the committee meeting, included an analysis which assumed that adalimumab and etanercept were equally effective (while the responses for infliximab remained the same as in the original analysis). The decision model was re-run with alternative parameter values using the TIDI interface during the committee meeting to provide an immediate answer to the queries which had arisen. An additional scenario was considered where the cost of one of the interventions depended on the way of administering it. The committee also inquired about the effect of removing one of the studies from the meta-analysis on the outcomes of the decision model. There was a concern that the population in this study was not representative of the target population and thus may provide a biased estimate of effect. The authors of this manuscript, who attended the committee meeting (S.B., A.J.S.), were able to re-run the meta-analysis after removing the study (using the interactive interface) and then re-run the decision model using the updated effectiveness estimates.

The delay caused by running the model was not substantial; it took between 1 and 2 minutes to run the model and an additional 2 minutes to run the meta-analysis. The committee was able to continue their discussions while the model was executed. However, due to the complexity of this particular model, only the deterministic version was used in real-time (the stochastic model could take several hours to run). The results were later confirmed by running the full probabilistic model; which we were confident would be the case since we had used the TIDI interface to monitor the error (which was small) across all pre-prepared scenarios prior to the committee meeting. This subsequent analysis was performed only for the scenario considering equal effectiveness of etanercept and adalimumab, and the remaining two additional scenarios mentioned above were not relevant to the decision.

Following the meetings, the authors surveyed the committee members about the usefulness of TIDI. Most of the responders were very positive about TIDI fulfilling its objectives of 1) giving support to the decision makers by allowing them to explore evidence syntheses and decision models under different scenarios and assumptions in real time, and 2) making the decision-making process faster and more efficient; while at the same time avoiding arbitrary limitations of the (pre-prepared) analysis that might compromise its validity. The committee found seeing the results helpful and although, in this particular appraisal, the answers to the committee's queries did not provide unexpected or decision-changing answers, the committee members appreciated that a lot of the time it is not clear what the effect of a change in assumptions would have on the outcomes. The responders to our survey also believed that it would be very desirable to make an interface like TIDI available routinely at NICE technology appraisal committee meetings. Although, not all committee members found it easy to understand the interface and the output it produced, all of the responders thought it would be desirable for the interface to integrate with the appraisal report in terms of presentation as well as content. We found that the members of the committee who were introduced to TIDI before the appraisal meeting were able to understand it better and appreciate it more than those who did not get such an introduction (due to time constraints only the briefest introduction was possible in the committee meeting). Overall we had a strong impression that TIDI can be a useful tool for the NICE committees, especially if the interface was introduced to the members in advance to make it easier for them to follow it during a dynamic discussion and if the interface is clearly readable (our font size was initially too small for the projection conditions).

## Discussion

TIDI has been created as a concept with the aim of helping the decision makers who are non-statistical experts, such as many members of the NICE appraisal committees, to make their decision process more transparent and efficient. It provides a tool to critique and explore the decision models and evidence syntheses (by which these models are informed) by a wider community of decision makers, not only those familiar with specialized software packages such as R and WinBUGS. It potentially allows the user to have in-depth access to and control over values/distributions on all model parameters and modeling assumptions, and at the same time uses flexible, clear, and hence transparent model components developed in R and WinBUGS. By making it possible to run models in real time, it makes the decision process more efficient. Being able to re-run a model under new scenarios in real time not only allows sensitivity analysis that potentially can change the final decision but also provides reassurance that, for example, uncertainty about a particular parameter does not have much effect on the cost-effectiveness estimates. Any required additional model scenarios or the interpretation of evidence can be considered during the committee meetings without the need for the committee to delay a decision and reconvene. It also allows an integrated analysis even when components of the modeling have been carried out in different packages.

As part of the process for developing a TIDI interface for use in “real-time,” we investigated the speed of varying software configurations. For illustration, the Anti-D stochastic model implemented in WinBUGS, when executed from TIDI (i.e., the version we have made available with this article), takes about 1.5 minutes to run 5000 iterations (without loss of speed compared to running the model directly from R using R2WinBUGS). It takes about 20 minutes to execute 5000 iterations of the same model developed entirely in Excel. The same model, implemented in R only, runs 5000 iterations in about 1.5 minutes. Hence we believe speed should also be a factor when deciding which combination of packages to use and significant speed improvements can be gained from using WinBUGS or R over using Excel for the whole application.

TIDI can be developed further to accommodate more complex decision models. For example the interface can have more options built into the “SetupAndRun” front page reflecting alternative assumptions about the model structure. Option buttons may allow the user to switch between different ways of modeling utility and cost over time, alternative parametric ways of modeling survival data or various alternative options defining stopping rules in Markov decision models, depending on the needs and complexity of the economic model. The part of the interface displaying the model results can also be designed in a number of ways depending on the needs of the decision makers. For example, when decision models are designed to compare more than two interventions, the ICERs resulting from the final selection of the most effective strategy (following exclusion of strategies being dominated or extendedly dominated [28]) can be displayed and also presented graphically on the cost-effectiveness plane (cost vs. QALY) by showing which strategy was selected as most effective and marking the strategies that have been excluded as being extendedly dominated by another strategy. This approach has been adopted by the authors in a NICE appraisal of the use of TNF-alpha inhibitors in treatment of psoriatic arthritis [26].

There are some limitations to TIDI that can be explored through further work. For example the influence analysis is limited when more than two strategies are being compared and in such cases tornado plots cannot provide definitive answers as they represent a single outcome (such as ICER or INMB) comparing only two interventions. To compensate for this limitation tornado plots for pair wise comparisons can be used to help to shed some light on what is important in the decision model. Further methodological considerations should aim to develop adequate tools equivalent to tornado plots which would address these limitations. Influence analysis not only assists in establishing which parameters can have the strongest impact on the estimates of the decision model, but potentially it can also be used to recommend further research in order to reduce uncertainty of some key parameters. To this end, it would be possible to include value of information calculations [29] within TIDI.

It may not be possible to use TIDI to run much more complex models (than the one used as an example here) in real time as it may take too much time to execute them. One solution to this problem, which was adopted in the NICE appraisal of the use of anti-TNFs in treatment of psoriatic arthritis [26], is to run only a deterministic model in real time. Obtained results can later be confirmed by the full stochastic model. Further work needs to be carried out to develop optimization methods for complex models so that it is possible to run them in real time during committee meetings. There is a lot of scope for further development of TIDI, for example it could include interactive tools for elicitation of expert opinions relating to bias in the meta-analysis module (as currently programmed for the anti-D example only existing elicitations can be incorporated). The meta-analysis module could also contain a tool for detecting and adjusting for publication bias and displaying funnel plots [30], and also for incorporation of empirical estimates of bias [31].

There are some technical challenges regarding the installation of the interface. This is due to the need to install multiple pieces of software in specific directories (as described in Appendix A). Our primary aim has been to provide a “proof of concept” integrated interactive approach to decision modeling, produce two working examples, and use one of those examples in a “real life” situation. As it stands, TIDI is not a consumer product (and we are not guaranteeing the application available is without bugs), but, since the only piece of software used which is not freeware is the widely used Excel, the approach should not be financially prohibitive. Although further development to produce a software program for the front end to Excel allowing the creation of TIDI interfaces without the need to program in VBA would be desirable, it would be a major undertaking; if it were to have desirable levels of flexibility. Therefore, a successful application of such an interface will require some (additional) programming skills from those conducting HTA, but we hope our illustrative example will demonstrate much of what is required. We also hope to secure funding to enable us to produce tutorial material in the near future. While we, the analysts, operated TIDI at the pilot NICE appraisal committee, we believe the interface was straight forward enough that a committee member could have done the job with less than 1 hour of training.

There are many advantages of a TIDI-type approach to decision modeling over the non-integrated two-step approach and the integrated, but not interactive, one-step approach described in the introduction. We believe that producing an interactive graphical representation of results of these usually complex analyses using a user-friendly Excel interface provides added value to the decision models by facilitating in-depth access to the details of the model structure to the decision makers and other stakeholders, adding to the transparency of the decision-making process.
