# Supplementary material for: Development of a Transparent Interactive Decision Interrogator to Facilitate the Decision-Making Process in Health Care
Source: Value Health. 2011 Jul;14(5):768–76. doi: 10.1016/j.jval.2010.12.002 (PMC3161376; doi:10.1016/j.jval.2010.12.002)
Supplement: Appendix A [file mmc1.doc]

**Appendix A**

**Instructions for installation of TIDI**

All the files required in order to execute TIDI are available at [http://www2.le.ac.uk/departments/health-sciences/research/ships/staff/sb309](https://securewebmail.le.ac.uk/owa/redir.aspx?C=fd7a234678704eeca9f21d788e4edf16&URL=http%3A%2F%2Fwww2.le.ac.uk%2Fdepartments%2Fhealth-sciences%2Fresearch%2Fships%2Fstaff%2Fsb309)

*© Copyright University of Leicester 2010*

*The software is intended for non-commercial purposes*

The TIDI interface developed works in conjunction with multiple packages and add-ons which need to be correctly installed on a PC before it can be used. The only commercial software required is Excel. Instructions for installing all other software, via Web download, are provided below. In addition you will need the specific TIDI interface files ([TIDIforAntiDmodel.xlsm](https://zdrive.le.ac.uk/s/sb309/BayesHTA/AntiD/ToSend/TIDIforAntiDmodel.xlsm), [antiDcorrelations.r](https://zdrive.le.ac.uk/s/sb309/BayesHTA/AntiD/ToSend/antiDcorrelations.r), [antiDdeterministicPlus.r](https://zdrive.le.ac.uk/s/sb309/BayesHTA/AntiD/ToSend/antiDdeterministicPlus.r), [antiDprobabilistic.r](https://zdrive.le.ac.uk/s/sb309/BayesHTA/AntiD/ToSend/antiDprobabilistic.r), [bias.adjust.tidi.v3.R](https://zdrive.le.ac.uk/s/sb309/BayesHTA/AntiD/ToSend/bias.adjust.tidi.v3.R), [bias.plot.R](https://zdrive.le.ac.uk/s/sb309/BayesHTA/AntiD/ToSend/bias.plot.R), [forest.plot.orig.R](https://zdrive.le.ac.uk/s/sb309/BayesHTA/AntiD/ToSend/forest.plot.orig.R), [forest.plot.R](https://zdrive.le.ac.uk/s/sb309/BayesHTA/AntiD/ToSend/forest.plot.R), [get.data.tidi.R](https://zdrive.le.ac.uk/s/sb309/BayesHTA/AntiD/ToSend/get.data.tidi.R), [LogNormalPdf.r](https://zdrive.le.ac.uk/s/sb309/BayesHTA/AntiD/ToSend/LogNormalPdf.r), [NormalPdf.r](https://zdrive.le.ac.uk/s/sb309/BayesHTA/AntiD/ToSend/NormalPdf.r), [StochasticTornadoPlot.r](https://zdrive.le.ac.uk/s/sb309/BayesHTA/AntiD/ToSend/StochasticTornadoPlot.r), [TornadoPlot.r](https://zdrive.le.ac.uk/s/sb309/BayesHTA/AntiD/ToSend/TornadoPlot.r), [UniformPdf.r](https://zdrive.le.ac.uk/s/sb309/BayesHTA/AntiD/ToSend/UniformPdf.r), [deterministic-mod-final-plus.odc](https://zdrive.le.ac.uk/s/sb309/BayesHTA/AntiD/ToSend/deterministic-mod-final-plus.odc), [probabilistic-mod.odc](https://zdrive.le.ac.uk/s/sb309/BayesHTA/AntiD/ToSend/probabilistic-mod.odc), [Readme.docx](https://zdrive.le.ac.uk/s/sb309/BayesHTA/AntiD/ToSend/Readme.docx)) which should have been included in the zip file which contained this Readme.docx file.

**1.** Make sure you have Microsoft Excel installed on your PC (this interface was created using Excel 2007 but it can be run under Excel 2003), and that its settings enable macros.

**2.** Install R and RExcel: Download "RAndFriends" package from: <http://rcom.univie.ac.at/> (go to “Downloads” tab) and install.

(You will need to uninstall any old version(s) of R previously on the computer.)

**3.** Install WinBUGS:

Download WinBUGS and the key from <http://www.mrc-bsu.cam.ac.uk/bugs/welcome.shtml>

Follow the instructions at the aforementioned website which explains how to install and unlock WinBUGS with the key file.

**4.** To be able to run macros, set a library in VBA: in Excel go to VBA editor (for example by pressing Alt F11) go to "Tools", then "References" and tick "RExcelVBAlib". *(This is probably only needed if using Excel 2003 or writing a new macro, but it is best to check if this library is set.)*

**5.** Install R2WinBUGS: in the package R, at the command prompt, type "install.packages("R2WinBUGS")"

**6.** All the files which were included with these instructions should be saved together in the same directory.

**7.**  You will need to change the directory/path location in the R flies which invoke the WinBUGS package to the location where WinBUGS is installed on the PC. The files in question are: antiDdeterministicPlus.r, antiDprobabilistic.r and [antiDcorrelations.r](https://zdrive.le.ac.uk/s/sb309/BayesHTA/AntiD/ToSend/antiDcorrelations.r) (this can be done from within R or using a text editor),

e.g. Where the code says

bugs.dir<-"z:/WinBUGS14"

this needs to be changed to the directory where WinBUGS is installed on the PC being used. e.g. the default installation directory for WinBUGS is:

bugs.dir<-"c:/Program Files/WinBUGS14"

8. Once this is done, you can initiate TIDI by opening the file [TIDIforAntiDmodel.xlsm](https://zdrive.le.ac.uk/s/sb309/BayesHTA/AntiD/ToSend/TIDIforAntiDmodel.xlsm) in Excel. The manuscript “Development of a Transparent Interactive Decision Interrogator to facilitate the decision making process in health care” provides guidance on the basic use of TIDI.

**(Note:**  **Do NOT** make any changes in the “Internal Info” spreadsheet – it contains original data – if deleted or changed will cause TIDI not running properly if at all.

Graphs (forest plots or acceptability curves) will be displayed only after running the meta-analysis or probabilistic model respectively.)
