# Supplementary material for: Development of a Transparent Interactive Decision Interrogator to Facilitate the Decision-Making Process in Health Care
Source: Value Health. 2011 Jul;14(5):768–76. doi: 10.1016/j.jval.2010.12.002 (PMC3161376; doi:10.1016/j.jval.2010.12.002)
Supplement: Appendix B [file mmc2.doc]

**Appendix B**

**Data storage and accessibility**

The spreadsheet format of the interface allows the storage of data and information for different purposes. Excel worksheet “Internal Info” contains all data required to execute the decision model and the meta-analysis. But there are also other ways of storing information in TIDI. For example a comment can be attached to a worksheet cell that contains details of the origin of information about each model parameter, such as a publication. This is shown in Figure 2 for cell B16 which contains the proportion of subjects who are RhD negative in the population. Further information on the model parameters can be obtained by viewing the probability density function plots for each stochastic parameter. The plots are produced by R, and RExcel allows them to be displayed in Excel (the graph appears after pressing the “Display PDF” button) as in the example showed in Figure B1.


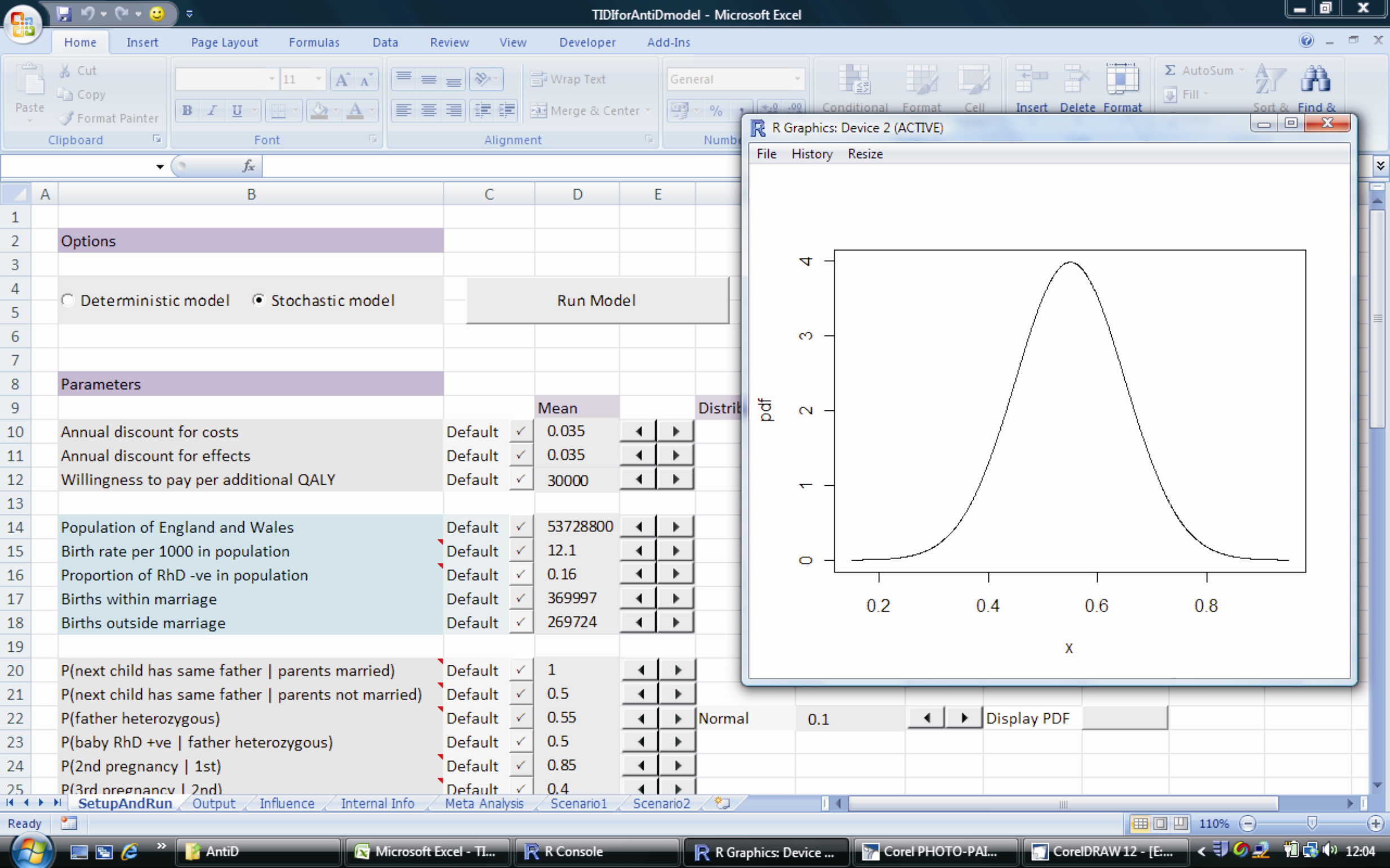
 **Figure B1** TIDI’s front page with probability density function plot.

Excel’s workbook structure also gives the possibility to record all of the model scenarios in the following way. The set of scenario parameter values and corresponding results can be saved in a separate worksheet “ScenarioN” (by pressing the “Save Scenario” button) creating a library of scenarios considered by decision-makers for later viewing. By pressing the “Reset Default Parameters” the base case parameter values can be restored. Furthermore, the whole set of parameter values and settings of any saved scenario can also be uploaded back to the “SetupAndRun” front page of the TIDI workbook (by pressing the “Load Scenario” button) for further considerations and amendments, after which the model can be re-run. This way all the scenarios considered by the analysts, such as a third-party academic group [27], preparing the appraisal report can be saved before the appraisal committee meeting. Any of those (including the base case scenario) can be used during the meeting, but also new scenarios can be built (on request from the committee members) by amending any of those pre-prepared and the new scenarios can be run in real time during the meeting and saved for reference or further considerations.

**Tornado plots**

An additional module of TIDI has been created to help to investigate the influence of individual parameters on the cost-effectiveness estimates from the decision model. To carry out the influence analysis, each parameter of the decision model needs to have a defined range of plausible values, for example a 95% confidence interval. The minimum and the maximum values of each parameter are used in the model one at a time, leaving the remaining parameters set to the same values as in a base case (reference) scenario. This gives the INMBs corresponding to the minimum and maximum values of each parameter and if those INMBs are far apart for a given parameter, it suggests that a change in this parameter will have a substantial impact on the final result of the decision model. Ranges of INMBs for each parameter are sorted and plotted from the widest on top to the narrowest on the bottom forming the tornado plot.


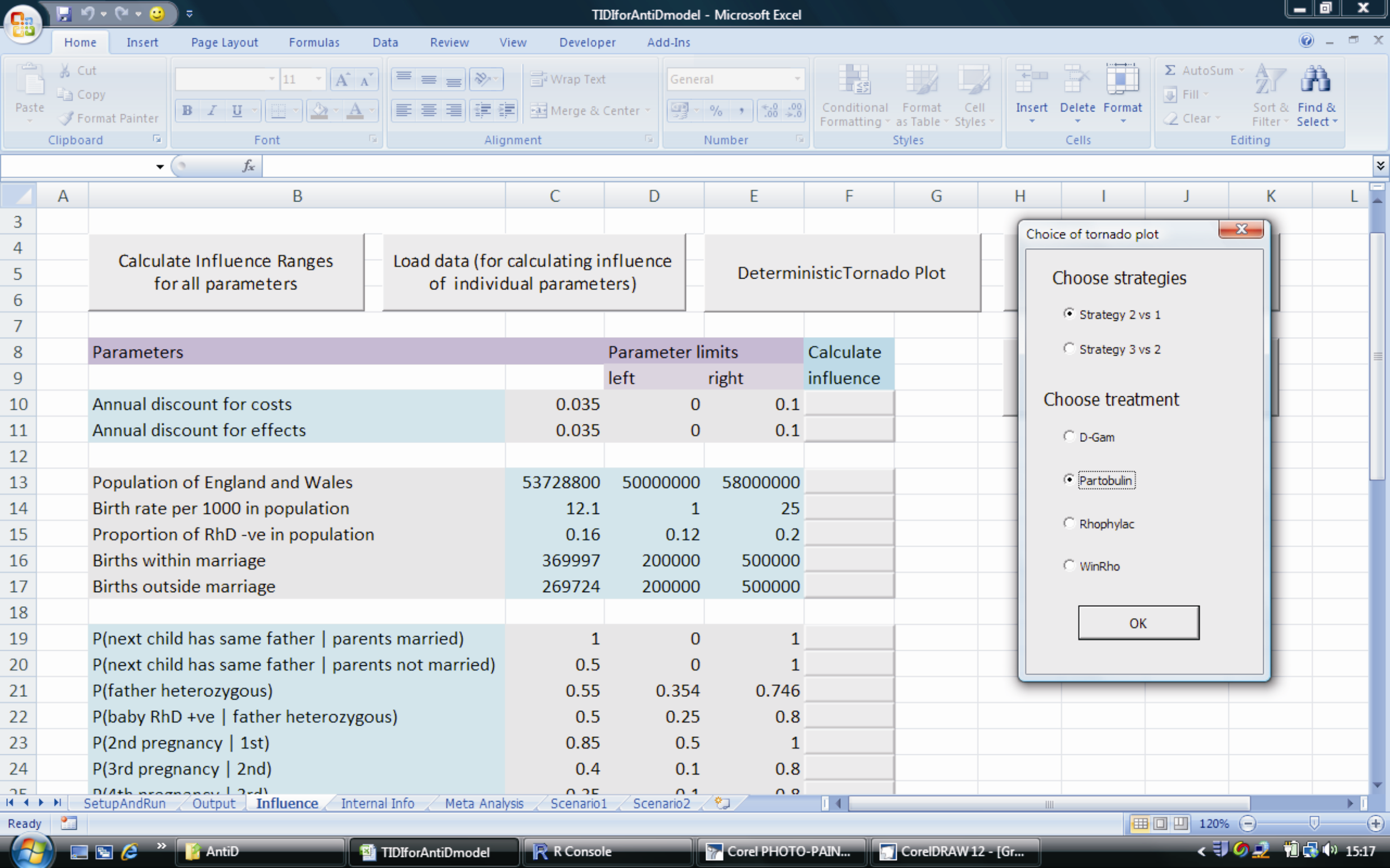


**Figure B2** Influence analysis spreadsheet contains the user menu for the choice of the tornado plot.

Figure B2 shows the “Influence” worksheet containing values and ranges of all the model parameters. Clicking on the “Tornado Plot” button shows a user form that allows the choice of a tornado plot for a given treatment and strategy comparison. Following this choice a tornado plot is generated by R and displayed in Excel as shown in Figure 5.


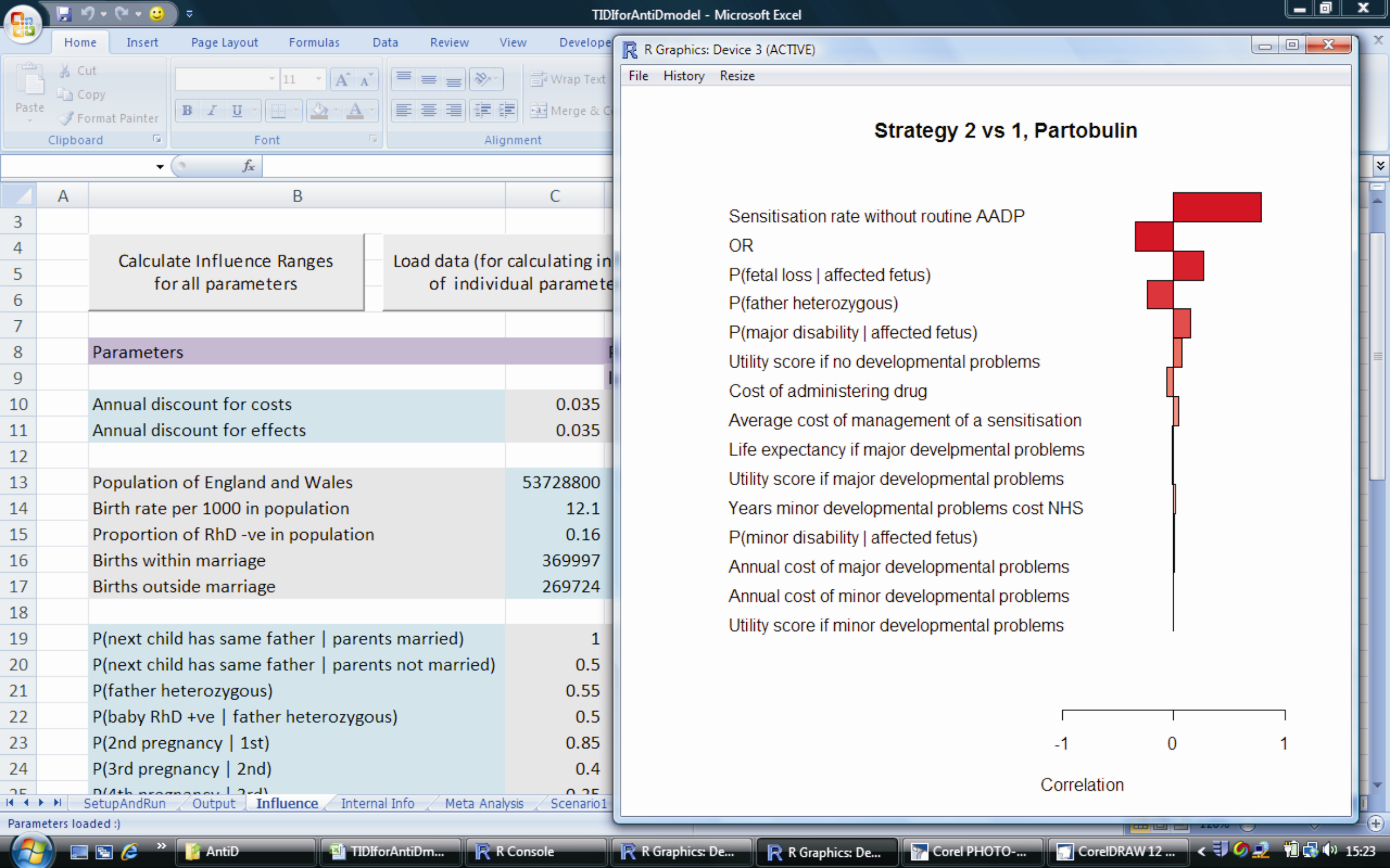


**Figure B3** Stochastic tornado plot for Partobulin and comparison of strategy 2 vs. 1.

Also a probabilistic approach to influence analysis can be adopted in which a stochastic version of a tornado plot can be explored. Such tornado plot displays correlations between each stochastic parameter and the outcome (INMB) as shown in Figure B3 (N.B. the decision model has to be run stochastically to use this feature). Here the parameters that are most highly correlated with the cost-effectiveness estimate are sensitisation rate without routine AADP and OR, which are the same parameters as in the deterministic tornado plot in Figure 5 if we disregard those with no uncertainty (marked on the plot by a star).
